# ADAMTS5-specific gapmer release from an albumin biomolecular assembly and cartilage internalization triggered by ultrasound

**DOI:** 10.1080/10717544.2025.2464921

**Published:** 2025-02-18

**Authors:** Marwa Elkhashab, Goncalo Barreto, Maxime Fauconnier, Yohann Le Bourlout, Laura B. Creemers, Heikki J. Nieminen, Kenneth A. Howard

**Affiliations:** a Interdisciplinary Nanoscience Center (iNANO) and Department of Molecular Biology, Aarhus University, Aarhus C, Denmark; b Medical Ultrasonics Laboratory (MEDUSA), Department of Neuroscience and Biomedical Engineering, Aalto University, Espoo, Finland; c Translational Immunology Research Program, University of Helsinki, Helsinki, Finland; d Orton Orthopedic Hospital, Helsinki, Finland; e Department of Orthopedics, University Medical Center Utrecht, Utrecht, The Netherlands

**Keywords:** Albumin, biomolecular assembly, Antisense, ultrasound, cartilage

## Abstract

**Objective:**

Antisense oligonucleotides (ASOs) have reached the clinic; however, they lack tissue specificity. Albumin is a plasma-abundant macromolecule that has been shown to accumulate in inflamed tissues. In this work, we have designed a recombinant human albumin (rHA)-based biomolecular assembly incorporating a DNase-resistant phosphorothioate-based complementary oligonucleotide (cODN) and an anti-ADAMTS5 ASO for potential delivery to inflamed sites. Ultrasound (US) was used to trigger ASO release from the assembly and enhance internalization into articular cartilage.

**Methods:**

A phosphorothioate cODN was conjugated to rHA through a maleimide cross-linker after which, a therapeutic ADAMTS5-specific gapmer ASO was annealed to the cODN. ASO release was assessed after exposing the biomolecular assembly to different US conditions using an US-actuated medical needle operating at 32.2 kHz. Gene silencing efficiency of US-treated anti-ADAMTS5 ASO was assessed in human primary chondrocytes isolated from osteoarthritic patients. US-mediated ASO penetration into articular cartilage was assessed on *ex vivo* bovine articular cartilage.

**Results:**

ASO release was observed after exposure to US waves in continuous mode conditions that did not compromise ASO gene silencing efficiency in human chondrocytes. Furthermore, US increased ASO internalization into bovine articular cartilage after 30 min of application without detrimental effects on chondrocyte viability.

**Conclusion:**

A medical needle driven by continuous US waves at 32.2 kHz has the capability of disassembling a duplex oligonucleotide and enhancing released ASOs internalization into articular cartilage. This work offers the potential delivery and the local triggered release of ASOs at the surface of articular cartilage providing potential benefits for the treatment of diverse cartilage pathologies.

## Introduction

Antisense oligonucleotides (ASOs) represent promising therapeutic agents within the field of gene therapy. They have reached the clinic for various medical conditions (Rinaldi & Wood, 2018), however, marketed ASOs depend on passive accumulation in the target tissue (Egli & Manoharan, 2023) which can lead to off-target accumulation and consequent adverse effects. To actively target the site of action, a ligand such as antibodies and small molecules which engage with the target tissue is required (Muro, 2012). However, active targeting poses a complex scale-up manufacturing process that presents hurdles for clinical translation (Rosenblum et al., 2018). As an alternative approach, disease pathophysiology such as disrupted vasculature endothelium associated with inflammation can be exploited to promote passive accumulation of specific drugs. It has been shown that during inflammatory pathogenesis, endothelial fenestrations become wider resulting in a preferential accumulation of macromolecules such as albumin (Crielaard et al., 2012). This phenomenon has been demonstrated in inflammatory arthitic diseases such as rheumatoid arthitis (RA) where albumin’s passive accumulation was further augmented by the increased tissue-catabolic needs (Wunder et al., 2003). Consequently, albumin represents a promising drug delivery system for inflammatory diseases like RA, endorsed by its inflamed tissue passive targeting capability, biocompatibility, biodegradability, low immunogenicity, and extended circulatory half-life (Larsen et al., 2016; Pilati & Howard, 2020).

We have previously introduced an ASO delivery platform based on an albumin-nucleic acid biomolecular assembly (Elkhashab et al., 2024). In this work, we have utilized an albumin oligonucleotide assembly incorporating a gapmer ASO through complementary base-pairing with a nuclease-resistant phosphorothioated (PS) oligonucleotide (cODN) to allow greater *in vivo* stability. Release of the ASO from the assembly, however, is a prerequisite for the ASO engagement with its target mRNA required for gene silencing. Therefore, ultrasound (US) was used in this study as a method for ASO-triggered release from the assembly (Supplementary Figure S1). Furthermore, this approach can be potentially used for site-specific ASO release exclusively at the target site of action, where the US waves are applied.

By virtue of important thermal and mechanical effects, US is considered a promising therapeutic instrument for its ability to induce tissue hyperthermia, and/or enhance internalization and release of drug payloads from delivery systems, facilitated by streaming and cavitation (Wang & Kohane, 2017). US waves used for drug release are above the audible limit as low as 20 kHz, even exceeding 1 MHz (Wang & Kohane, 2017). MHz US has been demonstrated to facilitate agent delivery into articular cartilage (Nieminen et al., 2017). Moreover, an US-actuated medical needle (USaMN) operating near 30 kHz (Le Bourlout et al., 2023) has been demonstrated to provide acoustic streaming and cavitation (Perra et al., 2021; 2022) as well as facilitate nanoparticle delivery in tissue phantoms (Perra et al., 2022). US has several therapeutic applications approved by the Food and Drug Administration (FDA) agency such as cancer tissue hyperthermia, glaucoma relief, kidney stone comminution and others (Miller et al., 2012).

Hence, this study presents a novel approach for albumin-oligonucleotide duplex biomolecular disassembly by utilizing US, which is a well-established technique with various clinical applications. US was investigated for triggering duplex disassembly and enhancing ASO penetration within articular cartilage.

## Materials and methods

### Oligonucleotide sequences

Recombinant human albumin wild type (rHA WT) was supplied by Albumedix Ltd (UK). Oligonucleotides (ODNs) were purchased from Integrated DNA Technologies (IDT^TM^). Sequences of ASO (Table 1) and primers (Table S 1) were taken from (Garcia et al. 2019)

**Table 1.** Oligonucleotides sequences used (*= phosphorothioate, + = locked nucleic acid (LNA)).

ADAMTS5-specific ASO	5’-NH_2_ +C*+T*+T*T*T*A*T*G*T*G*G* G*+T*+T*+G
cODN (PO)	5′- NH_2_ - CA ACC CAC ATA AAA G
cODN (PS)	5′- NH_2_ – C*A*A*C*C*C*A*C*A*T*A*A*A*A*G
Mismatch ASO	5′ +G*+G*+A*A*A*C*A*T*C*G*A*C*+A*+G*+T

### Cell culture and cartilage preparation

Human articular chondrocytes were isolated from articular cartilage obtained from three different OA patients undergoing total knee arthroplasty. The anonymous use of redundant tissue (Tissue removed during surgery as opposed to tissue removed for the purpose of research) for research purposes is part of the standard treatment agreement with patients in the University Medical Center Utrecht (UMCU) and was carried out under protocol n° 15–092 of the UMCU’s Review Board of the BioBank for the joint tissues. No consent is required by patients undergoing total knee arthroplasty at the UMCU, since they are informed of the potential usage of their removed tissue for research and education, however, the patient reserves the right to decline by completing a signed refusal form. During arthroplasty, both articular surfaces and the underlying bone are removed to allow for insertion of the prosthesis. The discarded tissue is then collected, and cartilage is isolated.

Tissue was immediately processed upon collected from surgery. Primary chondrocytes isolation was performed using type II collagenase as described by Garcia et al. (Garcia et al., 2019). Cartilage was removed from the subchondral bone and minced. Tissue fragments were sequentially digested at 37 °C in 0.2% (w/w) pronase (Roche Diagnostics) in DMEM(1X) + GlutaMAXTM-I (+ 4.5 g/L D-glucose, + pyruvate) (Gibco, USA) plus 100 U/ml penicillin/streptomycin (Gibco) for 2 hours, and in 0.075% collagenase type 2 (Worthington Biochemical, source: Clostridium histolyticum) in DMEM plus 200 U/mL penicillin/streptomycin overnight. Undigested debris was removed using a 70 μm cell strainer (Roth, Germany) followed by a PBS wash. Cells were cultured in a humidified incubator at 37 °C in Dulbecco’s Modified Eagle Medium; DMEM medium (with 4.5 g/L D-Glucose + Pyruvate) (Gibco, cat# 31966-021) with 10% fetal bovine serum (FBS, Gibco, cat# 10500-064), 100 U/ml penicillin/streptomycin (P/S, Gibco, cat# 15140-122), 0.2 nM L-ascorbic-2-phosphate sesquimagnesium salt hydrate (TCI EUROPE, cat # TCIAA2521) and 1 ng/ml basic fibroblast growth factor (bFGF, R&D Systems, cat# 233-FB/CF). Medium was renewed every 3 days. Cells were expanded until passage one and either frozen or further expanded and used for experiments at passage 2-4. Cells were seeded in DMEM medium (with 4.5 g/L D-Glucose + Pyruvate) supplied with 4 g/L human serum albumin (HA) (Sigma-Aldrich, cat# A1887), 1 × Insulin-Transferrin-Selenium-Ethanolamine (ITS-X, Gibco, cat# 51500056) and 100 U/ml P/S.

*Ex vivo* cartilage specimens were obtained from bovine knees of 3 animals from a local meat refinery (Heikin Liha Oy, Helsinki, Finland) within 6 days postmortem to ensure the preservation of chondrocyte viability. The knee cartilage was cut into 5 × 5 mm specimens containing both articular cartilage and 1-3 mm of underlying bone using a bone saw. The specimens were maintained in Dulbecco’s Modified Eagle Medium/Nutrient Mixture F-12 (DMEM/F-12, Gibco, cat# 21041025) with 10% FBS (Cytiva, cat# SH30088.03HI) and 1% P/S (MP Biomedicals, cat# 091670246) at 37 °C and 5% CO_2_.

### Polyacrylamide gel electrophoresis

Native polyacrylamide gels were prepared by combining ProtoGel (National Diagnostics, cat# EC-890), MilliQ water, 10X tris, borate, ethylenediamine tetraacetic acid (EDTA) (TBE, Invitrogen, cat# 15581-044), 10% ammonium persulfate (APS, Sigma-Aldrich, cat# A3678), and N,N,N’,N’-tetramethylethylenediamine (TEMED, Sigma-Aldrich, cat# T9281). It was then poured into 1 mm gel cassettes (Invitrogen, cat# NC2010) and allowed to polymerize by incubating at room temperature for 45 min.

Samples were mixed with loading buffer to achieve a glycerol concentration of 10% (VWR, cat# 24388.295) and Orange G at a concentration of 1 g/L (Sigma-Aldrich, cat# O3756). The gel was run in TBE buffer at 150 V for a duration of 30 to 90 min using an EPS 601 electrophoresis power supply (Amersham Biosciences). Following electrophoresis, the gel was stained with 1 X SYBR Gold (Invitrogen, cat# S11494) or 1 X SYBR GREEN II RNA gel stain (Invitrogen, cat# S7564) for 15 min. Visualization was performed using the Gel Doc EZ Imager (BioRad), while Amersham typhoon 5 (Cytiva) was utilized for both SYBR Gold and fluorescence imaging.

### Biomolecular assembly production

5′-amino modified ODN was mixed with 50 molar equivalent of cross-linker succinimidyl-[(N-maleimidopropionamido)-octaethyleneglycol] ester (SM(PEG)_8_, Sigma-Aldrich, cat# 746207) in dimethyl sulfoxide (DMSO, Sigma-Aldrich, cat# 34869) and 0.1 M 4-(2-hydroxyethyl)-1-piperazineethanesulfonic acid (HEPES, Sigma-Aldrich, cat# H4034) pH 8 overnight at 650 rpm, room temperature (RT). The activated ODN was then allowed to react with the thiol group of rHA in 0.1 M HEPES pH 7 overnight at 650 rpm at RT. The maleimide linkage was hydrolyzed afterwards, by mixing with Trizma^®^ base (tris base, Sigma-Aldrich, cat# T1503) buffer pH 9 overnight at 37 °C. The formed conjugate is then purified with HPLC. The conjugate was annealed to the ASO at a 1:1 molar ratio in 200 mM potassium acetate (Sigma-Aldrich, cat# P1190) and further purified with HPLC.

The biomolecular assembly protein concentration was determined by a Bicinchoninic Acid (BCA, Thermo Scientific, cat# 23225) assay. Briefly, 10 ul of the protein sample was mixed with 200 ul of the BCA reagent mixture and incubated at 37 °C for 30 min. The absorbance was then measured using a Clariostar plate reader at 562 nm.

Dual fluorescent labeled biomolecular assembly was produced as previously described (Elkhashab et al., 2024). Briefly, ASO was Cy5.5 labeled at the amino-modified 5′-end by mixing the ASO with N-hydroxy succinimide (NHS)-modified Cy5.5 fluorophore overnight at pH 8, 650 rpm and RT. The labeled ASO was then purified using reversed-phase HPLC.

Albumin was labeled nonspecifically at the lysines by mixing albumin with NHS-modified Cy3 fluorophore (Lumiprobe, cat# 21020) overnight at pH 8, 650 rpm and RT. Excess fluorophore was removed by spin filtration using a 10 kDa cutoff membrane filter (Amicon Ultra, Merck Millipore Ltd., cat# UFC501096).

Both the labeled albumin and ASO were then mixed in annealing buffer (200 mM potassium acetate) at 1:1 molar ratio overnight and further purified by HPLC.

### HPLC purification

The conjugate and the assembly were purified using a Mono Q 5/50 GL column (Sigma-Aldrich). The elution of the purified conjugate and assembly was performed by increasing the gradient of the elution buffer [(800 mM NaCl (Acros organics, cat# 207790010) for the conjugate, or 3 M NaCl for the assembly) and 20 mM Trizma hydrochloride (Tris HCl, Sigma-Aldrich, cat# T5941–500G)] over 25 min.

Cy5.5-modified ASO was purified using reversed-phase C-18 column (Xterra MS C18 5 μm). The ASO was loaded on the column using the loading buffer (5% TEAA (triethylammonium acetate, Sigma-Aldrich, cat# 69372) and 5% acetonitirile (VWR, cat# 83639.320)), while the elution buffer was composed of 100% acetonitrile.

### DNase-mediated ASO release

DNase I (DNA-free^™^ Kit TURBO^™^, Invitrogen, cat# AM1907) was incubated at 1 U/sample (0.5 µl) with 200 pmols of rHA-cODN(PO)/ASO and rHA-cODN(PS)/ASO assembly in 0.1 volume 10 x TURBO DNase Buffer at 37 °C for 30 min. The sample was then run on an 8% Native polyacrylamide gel at 150 V for 30 min and visualized after SYBR Gold staining.

### Ultrasound application

We employed an USaMN equivalent to that described by Le Bourlout *et al* (Le Bourlout et al., 2023). USaMN consists of a hypodermic needle (21 gauge, outer diameter = 0.8 mm) driven by a Langevin transducer operating at 32.2 kHz. A coupling waveguide is designed in a way that the longitudinal waves produced by the transducer are converted within a waveguide into flexural waves, when reaching the needle. Within the needle standing waves are generated with maximum displacement at the bevel tip. The US signals generated (B&K Precision, 4053B) and amplified by a custom-made device are summarized in Table 2.

**Table 2.** Applied US parameters. For each of these four configurations, three different durations of sonication were investigated: 1, 6 and 16 min.

	High amplitude continuous mode	High amplitude burst mode	Low amplitude continuous mode	Low amplitude burst mode
Consumed electrical power	1.8 W	1.0 W	0.2 W	0.1 W
Duty cycle	100% (continuous)	52% (burst)	100% (continuous)	52% (burst)
Burst period	n/a	18 ms	n/a	18 ms
Driving frequency	32.2 kHz	32.2 kHz	32.2 kHz	32.2 kHz

### Evaluation of ultrasound effect on gapmer ASO functionality

The gapmer was exposed to US waves through an USaMN operating at 32.2 kHz at low amplitude continuous mode for 30 min. A gapmer silencing efficiency experiment was then performed. Briefly, primary chondrocytes obtained from knee replacement surgeries of osteoarthritic patients were seeded in a 24-well plate (Thermo Scientific, cat# 142475) at a density of 100,000 cells/well. After 24 h the cells were treated with gapmer and gapmer exposed to US at a concentration of 1 μM then incubated at 5% CO_2_, 37 °C for 72 h.

An RT-qPCR assay was performed where total RNA was isolated with TRIzol (Invitrogen, cat# 15596-026) and reverse transcribed to cDNA using High-Capacity cDNA Reverse Transcription Kit (Applied Biosystems, cat# 368814), according to manufacturer’s protocol employing a SimpliAmp Thermal Cycler (Applied Biosystems). The cDNA was amplified with LightCycler^®^ 480 SYBR Green I Master (Roche Life Sciences, cat# 04707516001) in a LightCycler^®^ 480 II instrument (Roche). The expression of ADAMTS5 gene and housekeeping genes (GAPDH and 18S) was analyzed with three PCR amplification steps. Ct values were obtained by Roche LightCycler^®^ 480 software 1.5 and relative gene expression was calculated with PfaffI method to account for primers’ efficiencies.

### Ex vivo cartilage viability following ultrasound application

Cartilage specimens were stabilized vertically on one side of a quartz cuvette using clay. The USaMN was placed at a distance of 3 mm from the cartilage surface, parallel to the cartilage surface. The bevel opening was facing the cartilage surface for the sonication period. Then the needle was operated at 32.2 kHz with a low amplitude continuous mode. As a positive control, cartilage was incubated at 50 °C for 20 min.

Afterwards, the cartilage specimens were immersed in propidium iodide solution 20 µg/ml (Invitrogen, cat# P1304MP) for 10 min, then washed with phosphate-buffered saline (PBS). The specimens were cryo-frozen using a tissue-freezing medium (Surgipath FSC22 clear, cat# 3801480).

Frozen specimens were cryosectioned vertically using a Leica CM1900 cryostat into 13 µm sections and mounted using Molecular Probes^™^ SlowFade^™^ Diamond Antifade Mountant (Invitrogen, cat# S36972) containing DAPI on glass slides. Slides were visualized using ZOE^TM^ Fluorescent Cell Imager (Biorad).

### Gapmer ASO internalization in bovine cartilage

The same setup used for the viability study was applied to the internalization study. The cartilage specimens were mixed with dually labeled rHA(Cy3)-cODN (PS)/ASO(Cy5.5) biomolecular assembly 100 nM and the chosen US conditions (32.2 kHz, low amplitude continuous mode) were applied for 30 min. For controls, the pre-US specimen was cartilage pre-exposed to US for 30 min then mixed with the dually labeled biomolecular assembly for 30 min, while the passive diffusion specimen was cartilage mixed with the dually labeled biomolecular assembly for 30 min without the application of US.

The specimens were cryo-frozen in tissue-freezing medium using dry ice. Vertical sections were obtained using the Leica CM1900 cryostat and mounted using Molecular Probes^™^ SlowFade^™^ Diamond Antifade Mountant on glass slides. The slides were imaged using Zeiss Confocal microscope LSM 700 (Carl Zeiss MicroImaging). Images were processed with Zeiss Zen Black 2012 edition.

### Statistical analysis

Gene fold changes in primary chondrocytes following exposure to ASO treatments were assessed using the non-parametric Friedmann statistical test with a 95% confidence interval. The Mann-Whitney U test was employed to determine differences in ASO release among various assemblies at 95% confidence interval. Statistical analyses were conducted using GraphPad Prism software version 9.5.0.

## Results

### Ultrasound mediated ASO release

US waves were applied to rHA-cODN(phosphodiester, PO)/ASO biomolecular assembly through an US-enhanced needle in a sealed P-200 pipette tip. The different US conditions applied include high and low amplitudes, as summarized in Table 2. Sampling was performed after 1 min sonication, then repeated after 6 and 16 min total sonication, so that 3 samples are taken along one experiment.

**Figure 1.**
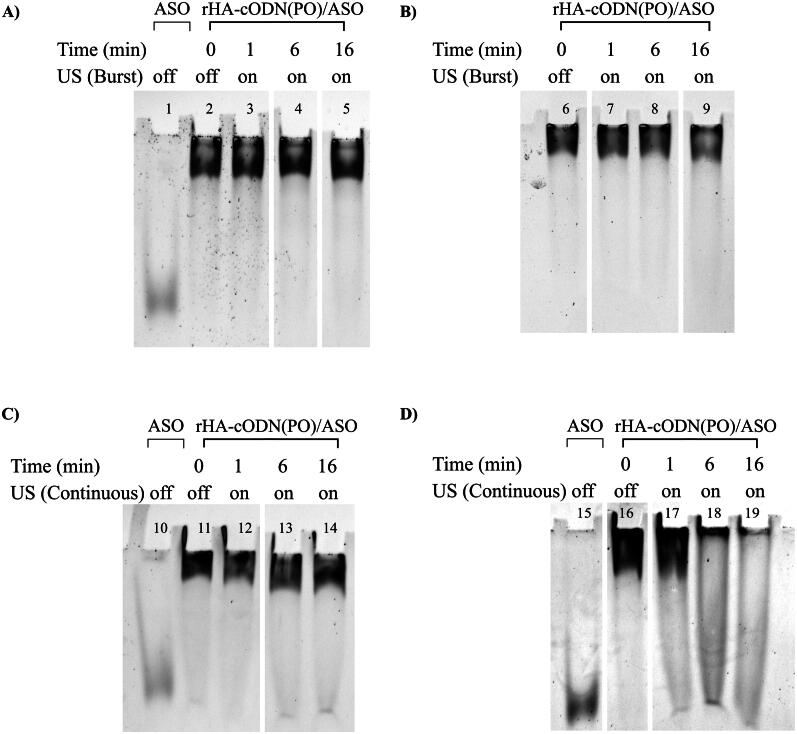
SYBR Gold staining of 8% native PAGE for the rHA-cODN(PO)/ASO biomolecular assembly after US exposure at different modes. A & B) Low and high amplitudes burst mode, respectively. C & D) Low and high amplitudes continuous mode, respectively. Lanes 1, 10 & 15: ASO, lanes 2-5, 6-9, 11-14 & 16-19: rHA-cODN(PO)/ASO biomolecular assembly. Gel images are focused on the relevant bands. Full gel images are provided in the Supplementary material.

Neither the low amplitude (Figure 1(A)) nor the high amplitude (Figure 1(B)) burst mode resulted in any observable release of the ASO with sonication duration up to 16 min.

However, the application of continuous low amplitude cycles resulted in a modest release of 1% of the ASO from the assembly after 6 min, and 6% after 16 min as shown in Figure 1(C) (lanes 13 and 14). When continuous waves at high amplitude were applied, the system experienced adverse consequences, including a noticeable elevation in temperature to the touch. Additionally, the polyacrylamide gel electrophoresis (PAGE) analysis revealed a smear pattern in the assembly, as evidenced in Figure 1(D) (lanes 18 and 19) indicative of protein denaturation. Therefore, the use of high amplitude settings was discontinued in this experiment.

**Figure 2.**
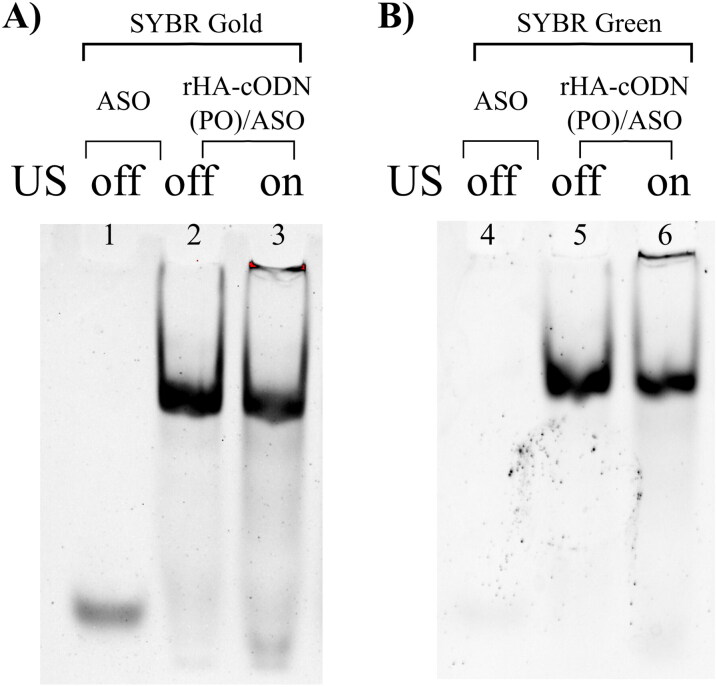
US-mediated ASO release (low amplitude continuous mode for 30 min). A & B) SYBR Gold and SYBR Green imaging of 8% native PAGE, respectively, lanes 1 & 2: ASO, lanes 2,3,5 & 6: rHA-cODN(PO)/ASO. Gel images are focused on the relevant bands. Full gel images are provided in the Supplementary material.

Consequently, the use of low amplitudes was chosen to proceed with later experiments. The use of low amplitude continuous mode for 30 min resulted in a noticeable release of the ASO from the assembly, evident by the presence of a distinct band representing free oligonucleotides in the lower region of the gel (Figure 2(A), lane 3)

Moreover, SYBR Green staining (indicative of double stranded DNA) of the US-treated rHA-cODN(PO)/ASO assembly showed a lower level of staining of the released oligonucleotides (in the lower region of the gel, Figure 2(B)) in comparison to SYBR Gold staining (Figure 2(A)) indicating the single-stranded nature of the released oligonucleotide.

**Figure 3.**
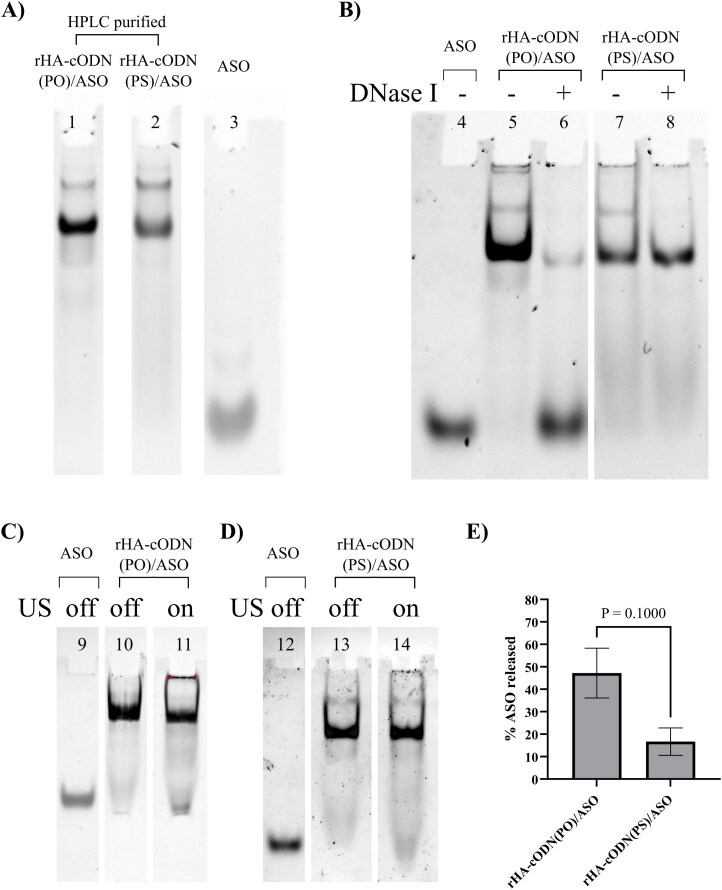
PAGE Showing the different biomolecular assemblies after HPLC purification and effect of DNase and US (low amplitude continuous mode for 30 min) on the ASO release. A) SYBR Gold staining of 8% native gel of the different assemblies. Lane 1: rHA-cODN(PO)/ASO, lane 2: rHA-cODN(PS)/ASO, lane 3: ASO. B) SYBR Gold staining of 8% native gel showing the effect of DNase on the release of ASO from different assemblies. Lane 4: ASO, lane 5 & 6: rHA-cODN(PO)/ASO, lane 7 & 8: rHA-cODN(PS)/ASO. C & D) SYBR Gold staining of 8% native gel showing the effect of US on rHA-cODN(PO)/ASO and rHA-cODN(PS)/ASO, respectively. Lane 9 & 12: ASO, lane 10 & 11: rHA-cODN(PO)/ASO, lane 13 & 14: rHA-cODN(PS)/ASO. E) Bar chart showing the percentage of ASO released after US application. Data were analyzed using ImageJ 1.53k, bar chart was created by GraphPad prism software, and error bars are the standard deviations of 3 independent experiments (*N* = 3). Statistical analysis was performed using Mann-Whitney U test using GraphPad prism software 9.5.0. Gel images are focused on the relevant bands. Full gel images are provided in the Supplementary material.

A DNase stable assembly comprising rHA conjugated to PS-modified cODN, to which the ASO was annealed (Figure 3(A)), was developed for further characterization under US conditions. The assemblies were purified using anion-exchange chromatography and the absence of free ASO was confirmed using native PAGE (Figure 3(A)) where no detectable free ASO bands were found in the lanes of the purified assemblies. Both the DNase-susceptible assembly (rHA-cODN(PO)/ASO) and the DNase-resistant assembly (rHA-cODN(PS)/ASO) were subjected to DNase I (Turbo^TM^). The DNase treatment resulted in a nearly complete release of the ASO from the rHA-cODN(PO)/ASO assembly, as illustrated in Figure 3(B). In contrast, no release of ASO was observed from the rHA-cODN(PS)/ASO assembly.

Noticeable ASO release was observed after applying low amplitude continuous mode US for 30 min. The ASO release levels averaged approximately 47.2% (confidence interval: 19.7%-74.8%) after applying US to the rHA-cODN(PO)/ASO assembly. On the other hand, lower ASO release averaging around 16.7% (confidence interval: 1.5%-31.8%) was observed after applying US to the rHA-cODN(PS)/ASO assembly, as shown in Figure 3(C–E).

### Ultrasound condition applied did not influence ASO functionality

A cellular gene silencing experiment was conducted to evaluate whether the gene silencing functionality of the ASO was maintained after US exposure.

**Figure 4.**
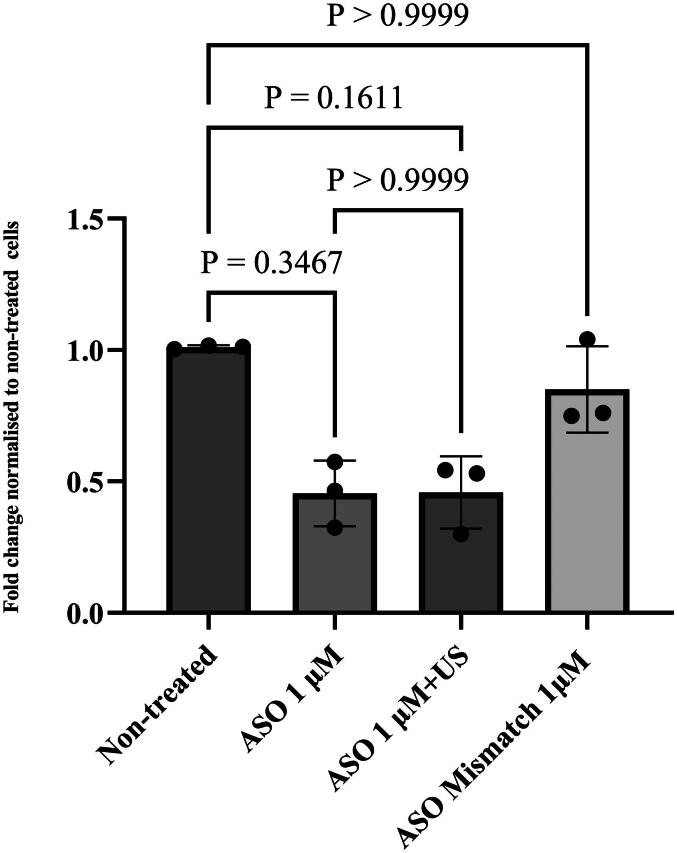
Bar chart showing the functionality of the ADAMTS5-specific ASO after exposure to US waves (low amplitude continuous mode for 30 min). Bar chart was created using GraphPad prism software 9.5.0 and error bars represent the standard deviations of data obtained from 3 independent experiments (*N* = 3) (each experiment was performed in triplicate). Each independent experiment was performed on primary human chondrocytes obtained from a different donor. Statistical analysis was performed using Friedman test using GraphPad prism software 9.5.0.

Approximately 54.2% ADAMTS5 gene silencing (confidence interval: 20.1%-88.4%) was observed with the US-treated ASO comparable to the silencing efficiency of 54.5% (confidence interval: 23.4%-85.7%) of the untreated ASO after a 72-h treatment period, as demonstrated in Figure 4.

### Ultrasound did not induce chondrocyte cell death in bovine cartilage

**Figure 5.**
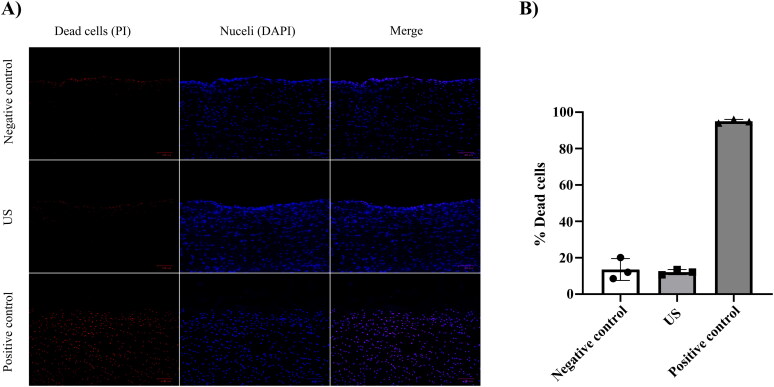
Biocompatibility of US conditions used on bovine cartilage. A) Images of bovine cartilage explants incubated in medium without US or heat exposure (negative control), exposed to US waves at low amplitude continuous mode for 30 min (US), or exposed to high temperature (50 °C) for 20 min (positive control). Dead cells were stained with propidium iodide (PI) (red), and nuclei were stained with DAPI (blue). Scale bar = 100 µm. B) Bar chart showing the quantified bovine cartilage chondrocytes bioavailability with the different conditions applied. Image analysis was performed using ImageJ of 3 different bovine knee joints (*N* = 3) (each knee joint was cut into three cartilage specimens; each cartilage specimen was cryosectioned into 3 different slices and three different images of the same slice were analyzed). Bar chart was created by GraphPad prism software, error bars are the standard deviations of data obtained from 3 different bovine knee joints.

The biocompatibility of the selected US conditions was evaluated on *ex vivo* bovine cartilage samples. Approximately 12% of chondrocytes were non-viable after 30 min of US application. This result was comparable to that observed in the non-treated cartilage specimens, which served as the negative control and exhibited approximately 13.5% non-viability, as illustrated in Figure 5(B).

The non-viable chondrocytes were primarily localized on the surface of the cartilage specimens in both the US-treated and non-treated specimens compared to the full-thickness non-viability observed in the heat-treated cartilage specimens, serving as the positive control, as shown in Figure 5(A).

### Ultrasound enhanced ASO internalization through bovine cartilage

**Figure 6.**
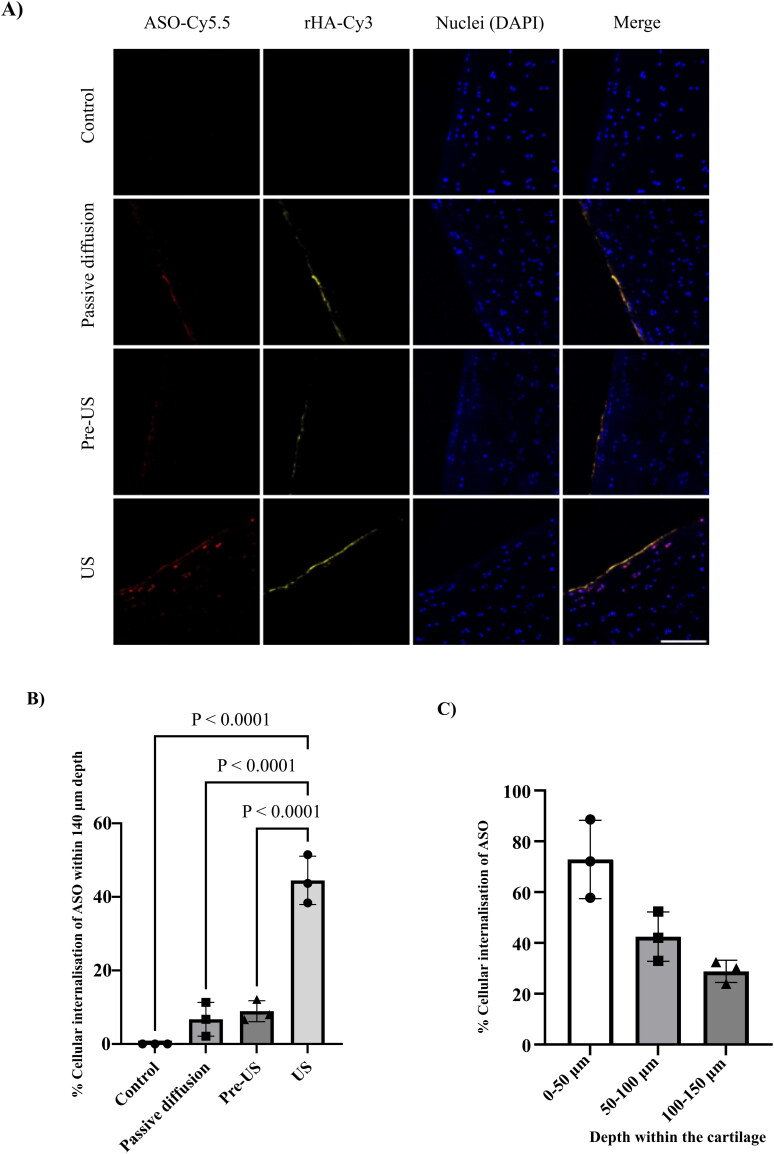
US-mediated ASO internalization in bovine cartilage. A) Confocal images showing the internalization of rHA (Cy3)-cODN(PS)/ASO(Cy5.5) biomolecular assembly within bovine cartilage. ASO(Cy5.5) is shown in red, rHA(Cy3) is shown in yellow and nuclei are stained with DAPI (blue). Scale bar = 100 µm. Control specimens represent tissue unexposed to US nor ASO. B) Bar chart showing quantified ASO(Cy5.5) internalization within 140 µm depth of bovine cartilage. C) Bar chart showing the differences of ASO internalization at different depth of bovine articular cartilage after US application. Image analysis was performed using ImageJ (one cartilage specimen was taken from each knee, each was cryosectioned into one slice and three different images of the same slice were analyzed). Statistical analysis was performed using GraphPad prism 9.5.0 software. One-way ANOVA statistical test was applied, error bars represent the standard deviations of data from 3 different bovine knee joints (*N* = 3).

The internalization of the rHA-cODN(PS)/ASO assembly into cartilage under US conditions was investigated using confocal microscopy. The images indicate that only the Cy5.5 labeled ASO appeared to be internalized deeper within the cartilage, whereas the Cy3 labeled rHA was primarily observed on the cartilage surface (Figure 6(A)).

After 30 min of incubation at room temperature (RT), approximately 6.7% of the chondrocytes located within the upper 140 µm depth of the cartilage exhibited colocalization with the ASO. Pre-sonication of the cartilage specimen did not significantly increase cellular internalization with only 8.9% of chondrocytes (within 140 µm depth of the cartilage) colocalized with Cy5.5 labeled ASO, and the ASO was mainly localized in superficial chondrocytes as shown in Figure 6(B).

However, when sonication was applied, the depth of ASO internalization was increased and the cellular internalization reached an average of 44.5% within the upper 140 µm depth of the cartilage, as shown in Figure 6(B). Furthermore, the internalization of the ASO after sonication was found to be higher in the superficial layer of the cartilage compared to the deeper layers, as shown in Figure 6(C).

## Discussion

Albumin has been utilized for passive targeting of drugs in inflammatory diseases including RA. For example, albumin-drug conjugates like albumin-methotrexate showed a higher effect on inhibiting arthitis development compared to methotrexate alone in murine models (Fiehn et al., 2004). In addition, an antisense NF-kappa B oligonucleotide encapsulated in albumin microspheres was able to reduce paw swelling in RA rats (Akhavein et al., 2009). Furthermore, the approval of the Ozoralizumab, a trivalent antibody with affinity to albumin, under the brand name of Nanozora^®^ in Japan for RA patients (Keam, 2023) promotes its clinical potential.

In previous work by our lab, we developed a DNase-susceptible rHA-oligonucleotide assembly as a carrier for an ADAMTS5-specific gapmer ASO to improve the circulatory half-life and lower renal accumulation (Elkhashab et al., 2024). In that work, ASO annealing to rHA-ODN conjugate was confirmed using fluorescence imaging of a native PAGE of the assembly, with minimal nonspecific binding between ASO and rHA shown. Although this assembly preserved gapmer functionality, *in vivo* stability was not investigated in the paper. In this work, we have modified the assembly by incorporating a PS-modified oligonucleotide conjugated to rHA, to which the ADAMTS5 gapmer ASO was annealed, in order to improve DNase resistance (Spitzer & Eckstein, 1988) for potential *in vivo* applications. However, the release of the ASO is required to allow mRNA engagement and gene silencing necessitating a strategy that could be used for triggering ASO release preferably at the site of action. Different physical sources, including light, heat, magnetic field, electric field and US, have been investigated for triggering local drug release (Wang & Kohane, 2017).

US has been shown to release payloads of non-gas-filled delivery systems, for example, an antibiotic molecule was released from a poly-aptamer system after the application of US which in turn caused the breakage of covalent and non-covalent bonds along the poly-aptamer (Huo et al., 2021). In this work, US was used to mechanically trigger the release of the ASO locally at the tissue of action, i.e. articular cartilage. The application of US waves in continuous amplitude mode to the rHA-cODN/ASO system triggered ASO release after 30 min of application. However, low amplitudes were used instead of high amplitudes, to reduce the possibility of the denaturation of rHA due to the resulting increase in temperature induced by the acoustic waves.

The use of a DNase-susceptible phosphodiester (PO) cODN in the assembly can potentially raise issues of *in vivo* instability due to premature ASO release in the circulation by the action of serum DNase. Therefore, a PS-modified cODN was used to replace the PO cODN in the assembly for further optimization of the US conditions. The use of PS-modified cODN confers resistance to nuclease actions (Crooke et al., 2020), which in turn potentially confers *in vivo* stability with minimal ASO release in circulation.

It was shown previously that US waves have the capacity to break hydrogen bonds in polymer solutions like polyacrylic acid and alumina slurry, in which the applied frequency and the output power were determining variables (Le Ngoc & Takaomi, 2010). US can degrade DNA duplex either by the breakage of hydrogen bonds between DNA bases or by the single or double-strand DNA rupture (Elsner & Lindblad, 1989). For instance, if the applied US conditions created inertial cavitation effect, i.e. the nucleation and unstable oscillation of micro (Yan et al., 2013) and nanobubbles (Abdalkader et al., 2017), free radicals can be created causing the rupture of DNA (Elsner & Lindblad, 1989). In our work, we suggest that the release of the ASO is due to the breakage of the hydrogen bonds between the ASO and cODN bases. This is supported by SYBR Gold staining intensity of the rHA-cODN band which did not show significant decrease after ASO release. Additionally, the ASO functionality remained intact following US application demonstrating a comparable gene silencing efficiency of 55% to the ASO non-exposed to US, which excludes the possibility of ASO degradation after US exposure. In addition, the single stranded nature of the released ODNs was investigated using post-electrophoretic SYBR Green staining, since SYBR Green preferentially binds double stranded ODNs. Following SYBR Green staining, the released ODNs showed faint bands which indicate the single-stranded nature of the released ODNs which may exclude the breakage of the linker and release of the duplex ODNs as a result of US application. Furthermore, maximum temperature increase achieved by US application at the conditions used reached around 37 °C, which in turn did not affect the duplex stability or trigger ASO release, when applied to the assembly without US. Despite the temperature remaining below 37 °C after US application, the 15 degrees-increase in temperature following 30 min of US application needs to be addressed for clinical applications such as temperature control techniques.

Interestingly, ASO release from rHA-cODN(PS)/ASO assembly was lower than that from rHA-cODN(PO)/ASO assembly. This result supports the observation that ASO release was not temperature-based since incorporating PS in the ODN backbone reduces the melting temperature of the duplex (Stein et al., 1988). It is unlikely that the re-annealing of the released ASO to the rHA-cODN(PS) conjugate after US application caused the observed decrease in ASO release from the rHA-cODN(PS)/ASO assembly, since no change in the percentage of released ASO was observed after re-analysing the US-exposed rHA-cODN(PS)/ASO assembly following overnight incubation. The reason for the lower ASO release observed from rHA-cODN(PS)/ASO assembly compared to that from rHA-cODN(PO)/ASO assembly, therefore, requires further investigation.

Depending on the range of frequency and amplitude applied, US waves can generate cavitation with potentially violent activity, which when operated in biological tissues, can result in tissue damage (Mancia et al., 2019). However, when US was applied to *ex vivo* bovine cartilage, no extensive damage to the chondrocytes throughout the cartilage tissue was observed. This may be explained by the low frequency used in USaMN resulting in bubble resonance size of > 100 µm, mitigating localized stress points that would be essential for tissue damage. Articular cartilage is dense tissue, which is not likely to permit bubble growth to the resonance size within extracellular space measured in nm scale. Therefore, the selected low frequency may be a risk mitigation for cartilage damage. Moreover, considering the potential *in vivo* applications, the USaMN enables the positioning of the sound source, specifically the flexurally oscillating needle tip, within the joint and facilitates penetration of the synovium. Therefore, USaMN can be considered as a potential modality to enhance payload internalization into the cartilage. Future work should investigate US-triggered-mediated delivery of ASO into cartilage in an animal model.

The application of US to bovine cartilage in the presence of labeled assembly, resulted in deep ASO internalization in the cartilage tissue, which could be due to the ASO transport by acoustic streaming caused by the primary acoustic field of the oscillating USaMN tip (Perra et al., 2022) or by shock waves arising from collapsing bubbles outside cartilage occurring subsequent to the US-mediated release from the assembly. There was no improvement in ASO internalization observed, when comparing US-treated cartilage to US-non-treated cartilage, when US was not employed simultaneously with the assembly. This suggests that the ASO internalization was enhanced by the application of US waves rather than any change in the cartilage tissue properties induced by the US application.

## Conclusion

This work introduces a novel method to trigger the disassembly of stabilized duplex DNA ODNs within an albumin-nucleic acid delivery system, that has potential applications for tissue-localized US-triggered release of therapeutic oligonucleotides at sites of inflammation.

## Supplementary Material

Supplemental Material

## Data Availability

The datasets used and/or analyzed during the current study are available from the corresponding author on reasonable request.

## Supplementary material

Additional data are provided as a supplementary file (named Supplementary material) in PDF format. The Supplementary file includes a table describing the sequences of primers used in the RT qPCR assay, in addition to a schematic drawing displaying the US experimental setup. Different gels showing the influence of used US conditions on the temperature and the release of the ASO from the assembly are included in the Supplementary material file. Full gels for Figures 1–3 are included.

## References

[CIT0001] Abdalkader R, Kawakami S, Unga J, et al. (2017). The development of mechanically formed stable nanobubbles intended for sonoporation-mediated gene transfection. Drug Deliv 24:320–7. doi:10.1080/10717544.2016.1250139. 28165819 PMC8241156

[CIT0002] Akhavein N, Oettinger CW, Gayakwad SG, et al. (2009). Treatment of adjuvant arthritis using microencapsulated antisense nf-κb oligonucleotides. J Microencapsul 26:223–34. doi:10.1080/02652040802268691. 18666015

[CIT0003] Crielaard BJ, Lammers T, Schiffelers RM, Storm G. (2012). Drug targeting systems for inflammatory disease: One for all, all for one. J Control Release 161:225–34. doi:10.1016/j.jconrel.2011.12.014. 22226771

[CIT0004] Crooke ST, Vickers TA, Liang XH. (2020). Phosphorothioate modified oligonucleotide-protein interactions. Nucleic Acids Res 48:5235–53. doi:10.1093/nar/gkaa299. 32356888 PMC7261153

[CIT0005] Egli M, Manoharan M. (2023). Chemistry, structure and function of approved oligonucleotide therapeutics. Nucleic Acids Res 51:2529–73. doi:10.1093/nar/gkad067. 36881759 PMC10085713

[CIT0006] Elkhashab M, Dilek Y, Foss M, et al. (2024). A modular albumin-oligonucleotide biomolecular assembly for delivery of antisense therapeutics. Mol Pharm 21:491–500. doi:10.1021/acs.molpharmaceut.3c00561. 38214218 PMC10848253

[CIT0007] Elsner HI, Lindblad EB. (1989). Ultrasonic degradation of DNA. DNA 8:697–701. doi:10.1089/dna.1989.8.697. 2693020

[CIT0008] Fiehn C, Müller-Ladner U, Gay S, et al. (2004). Albumin-coupled methotrexate (mtx-hsa) is a new anti-arthritic drug which acts synergistically to mtx. Rheumatology 43:1097–105. doi:10.1093/rheumatology/keh254. 15199219

[CIT0009] Garcia JP, Stein J, Cai Y, et al. (2019). Fibrin-hyaluronic acid hydrogel-based delivery of antisense oligonucleotides for adamts5 inhibition in co-delivered and resident joint cells in osteoarthritis. J Control Release 294:247–58. doi:10.1016/j.jconrel.2018.12.030. 30572032

[CIT0010] Huo S, Zhao P, Shi Z, et al. (2021). Mechanochemical bond scission for the activation of drugs. Nat Chem 13:131–9. doi:10.1038/s41557-020-00624-8. 33514936

[CIT0011] Keam SJ. (2023). Ozoralizumab: first approval. Drugs 83:87–92. doi:10.1007/s40265-022-01821-0. 36509938

[CIT0012] Larsen MT, Kuhlmann M, Hvam ML, Howard KA. (2016). Albumin-based drug delivery: Harnessing nature to cure disease. Mol Cell Ther 4:3. doi:10.1186/s40591-016-0048-8. 26925240 PMC4769556

[CIT0013] Le Bourlout Y, Ehnholm G, Nieminen HJ. (2023). Multi-modal transducer-waveguide construct coupled to a medical needle. J Acoust Soc Am 154:3388–96. doi:10.48550/arXiv.2203.14792. 37991464

[CIT2039532] Le Ngoc, N., Takaomi, K., (2010). Ultrasound stimulus effect on hydrogen bonding in networked alumina and polyacrylic acid slurry. Ultrason Sonochem 17 (1):186–192. doi:10.1016/j.ultsonch.2009.04.007 19464939

[CIT0014] Mancia L, Vlaisavljevich E, Yousefi N, et al. (2019). Modeling tissue-selective cavitation damage. Phys Med Biol 64:225001. doi:10.1088/1361-6560/ab5010. 31639778 PMC6925591

[CIT0015] Miller DL, Smith NB, Bailey MR, et al. (2012). Overview of therapeutic ultrasound applications and safety considerations. J Ultrasound Med 31:623–34. doi:10.7863/jum.2012.31.4.623. 22441920 PMC3810427

[CIT0016] Muro S. (2012). Challenges in design and characterization of ligand-targeted drug delivery systems. J Control Release 164:125–37. doi:10.1016/j.jconrel.2012.05.052. 22709588 PMC3481020

[CIT0017] Nieminen HJ, Barreto G, Finnilä MA, et al. (2017). Laser-ultrasonic delivery of agents into articular cartilage. Sci Rep 7:3991. doi:10.1038/s41598-017-04293-5. 28638116 PMC5479804

[CIT0018] Perra E, Hayward N, Pritzker KPH, Nieminen HJ. (2022). An ultrasonically actuated fine-needle creates cavitation in bovine liver. J Acoust Soc Am 151:3690–702. doi:10.1121/10.0010534. 35778205

[CIT0019] Perra E, Hayward N, Pritzker KPH, Nieminen HJ. (2022). An ultrasonically actuated needle promotes the transport of nanoparticles and fluids. J Acoust Soc Am 152:251–65. doi:10.1121/10.0012190. 35931509

[CIT0020] Perra E, Lampsijärvi E, Barreto G, et al. (2021). Ultrasonic actuation of a fine-needle improves biopsy yield. Sci Rep 11:8234. doi:10.1038/s41598-021-87303-x. 33859220 PMC8050323

[CIT0021] Pilati D, Howard KA. (2020). Albumin-based drug designs for pharmacokinetic modulation. Expert Opin Drug Metab Toxicol 16:783–95. doi:10.1080/17425255.2020.1801633. 32729729

[CIT0022] Rinaldi C, Wood MJA. (2018). Antisense oligonucleotides: The next frontier for treatment of neurological disorders. Nat Rev Neurol 14:9–21. doi:10.1038/nrneurol.2017.148. 29192260

[CIT0023] Rosenblum D, Joshi N, Tao W, et al. (2018). Progress and challenges towards targeted delivery of cancer therapeutics. Nat Commun 9:1410. doi:10.1038/s41467-018-03705-y. 29650952 PMC5897557

[CIT0024] Spitzer S, Eckstein F. (1988). Inhibition of deoxyribonucleases by phosphorothioate groups in oligodeoxyribonucleotides. Nucleic Acids Res 16:11691–704. doi:10.1093/nar/16.24.11691. 2850541 PMC339104

[CIT0025] Stein C, Subasinghe C, Shinozuka K, Cohen JS. (1988). Physicochemical properties of phospborothioate oligodeoxynucleotides. Nucleic Acids Res 16:3209–21. doi:10.1093/nar/16.8.3209. 2836790 PMC336489

[CIT0026] Wang Y, Kohane DS. (2017). External triggering and triggered targeting strategies for drug delivery. Nat Rev Mater 2:17020. doi:10.1038/natrevmats.2017.20.

[CIT0027] Wunder A, Müller-Ladner U, Stelzer EHK, et al. (2003). Albumin-based drug delivery as novel therapeutic approach for rheumatoid arthritis. J Immunol 170:4793–801. doi:10.4049/jimmunol.170.9.4793. 12707361

[CIT0028] Yan F, Li L, Deng Z, et al. (2013). Paclitaxel-liposome–microbubble complexes as ultrasound-triggered therapeutic drug delivery carriers. J Control Release 166:246–55. doi:10.1016/j.jconrel.2012.12.025. 23306023

