# Supplementary material for: ADAMTS5-specific gapmer release from an albumin biomolecular assembly and cartilage internalization triggered by ultrasound
Source: Drug Deliv. 2025 Feb 18;32(1):2464921. doi: 10.1080/10717544.2025.2464921 (PMC11841101; doi:10.1080/10717544.2025.2464921)
Supplement: Supplemental Material [file IDRD_A_2464921_SM9345.docx]

Supplementary material

ADAMTS5-specific gapmer release from an albumin biomolecular assembly and cartilage internalisation triggered by ultrasound

Marwa Elkhashab^1^, Gonçalo Barreto^2,3,4^, Maxime Fauconnier^2^, Yohann Le Bourlout^2^, Laura B. Creemers^5^, Heikki J. Nieminen^2^, Kenneth A. Howard^1*^

^1^ Interdisciplinary Nanoscience Center (iNANO), Department of Molecular Biology, Aarhus University, DK-8000 Aarhus C, Denmark.

^2^ Medical Ultrasonics Laboratory (MEDUSA), Department of Neuroscience and Biomedical Engineering, Aalto University, 02150, Espoo, Finland.

^3^ Translational Immunology Research Program, University of Helsinki, 00100, Helsinki, Finland.

^4^ Orton Orthopedic Hospital, Tenholantie 10, 00280, Helsinki, Finland.

^5^ Department of Orthopedics, University Medical Center Utrecht, 3584 CT Utrecht, The Netherlands

Table S 1 Sequences of primers used for RT-qPCR

| ADAMTS5 | Forward | 5’ GCCAGCGGATGTGTGCAAGC 3’ |
| --- | --- | --- |
|  | Reverse | 5’ ACACTTCCCCCGGACGCAGA 3’ |
| GAPDH | Forward | 5’ TGCACCACCAACTGCTTAGC 3’ |
|  | Reverse | 5’ GGCATGGACTGTGGTCATGAG 3’ |
| 18S | Forward | 5’ GTAACCCGTTGAACCCCATT 3’ |
|  | Reverse | 5’ CCATCCAATCGGTAGTAGCG 3’ |


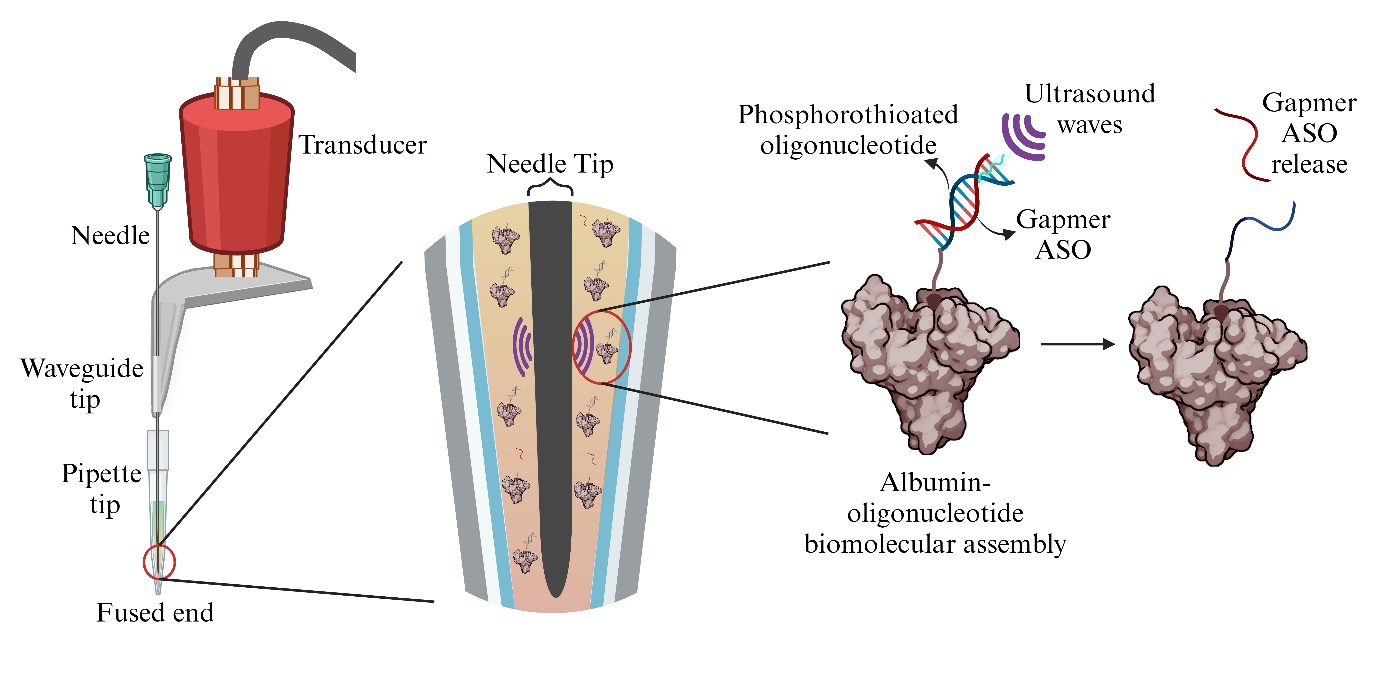


Figure S 1 Schematic representation of the experimental apparatus employing ultrasound for triggering ASO release from the biomolecular assembly. Figure was created using Biorender.com.


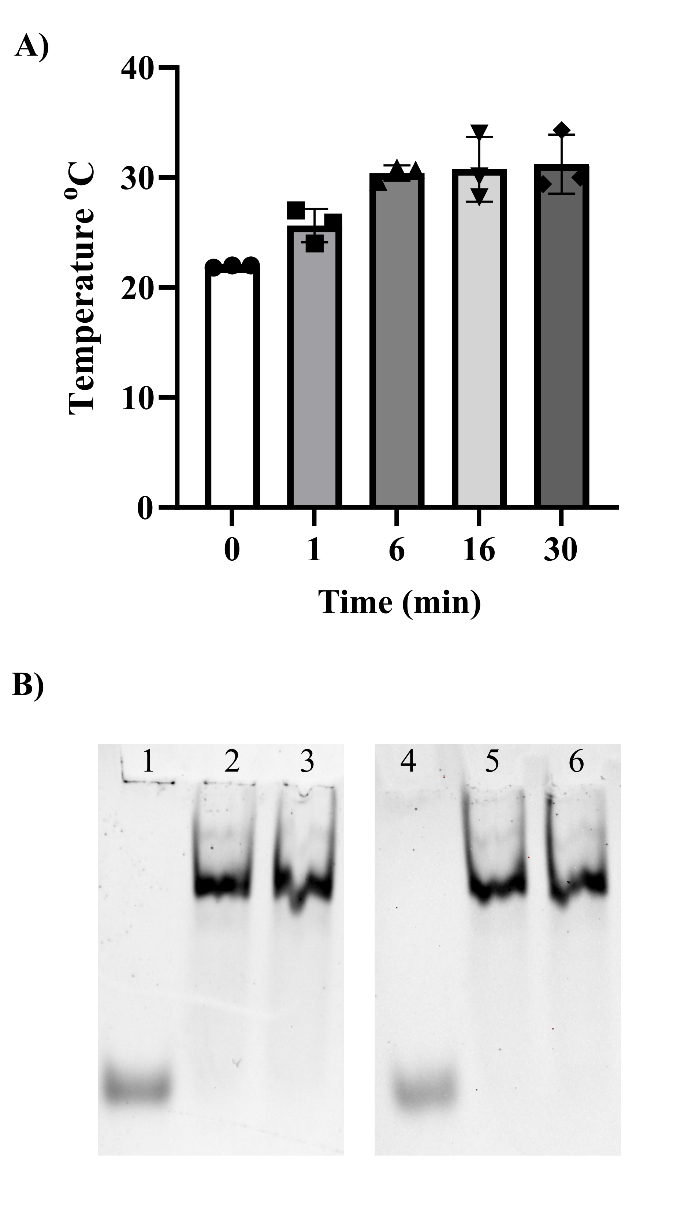


Figure S 2 US-mediated temperature increase during exposure to low amplitude-continuous mode US. A) Bar chart showing the gradual increase in temperature at the superficial surface of the sample in contact with the US needle. Bar chart was created by GraphPad prism software, error bars are standard deviations of 3 independent experiments. B) SybrGold imaging of 8% native gel showing the effect of temperature at 37^o^C on the release of the assembly. Lane 1: ASO, lane 2: rHA-cODN(PO)/ASO at RT, lane 3: rHA-cODN(PO)/ASO at 37^o^C for 30 min, lane 4: ASO, lane 5: rHA-cODN(PS)/ASO at RT, lane 6: rHA-cODN(PS)/ASO at 37^o^C for 30 min.


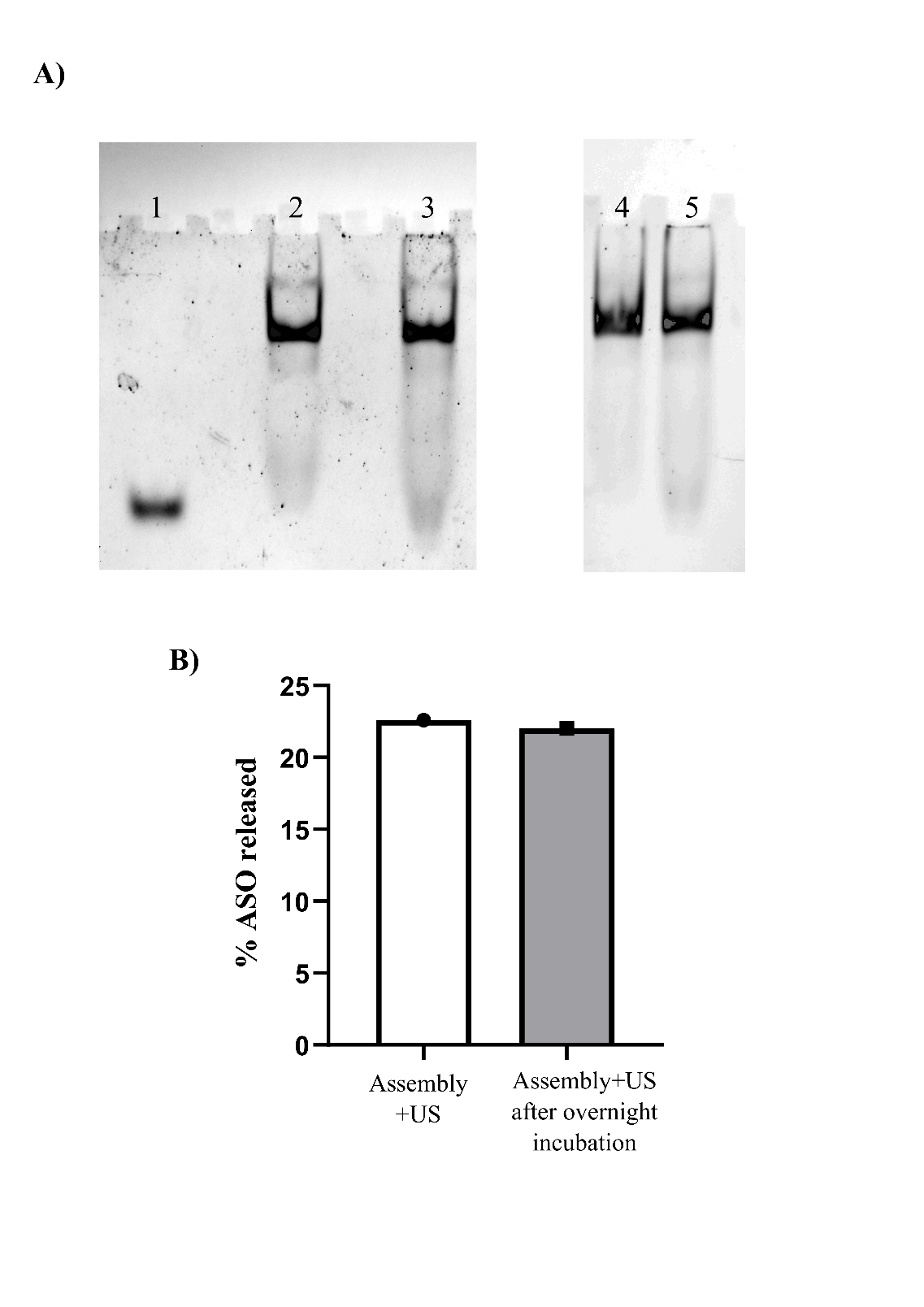


Figure S 3 Investigation of re-annealing possibility after US application. Following US application, the used assembly batch was incubated overnight at RT and re-run on a native polyacrylamide gel. A) 10% Native PAGE showing the ASO release from rHA-cODN(PS)/ASO assembly directly after US application. Lane 1: ASO, lane 2: rHA-cODN(PS)/ASO, lane 3: rHA-cODN(PS)/ASO + US, Lane 4: rHA-cODN(PS)/ASO, lane 4: rHA-cODN(PS)/ASO + US after overnight incubation. B) Bar chart showing the percentage of ASO released directly after US application and after overnight incubation. Data were analysed using ImageJ 1.53k and bar chart was created by GraphPad Prism software 9.5.0.

1. **B)**

ASO rHA-cODN(PO)/ASO rHA-cODN(PO)/ASO

Time 0 1 6 16 Time 0 1 6 16

US(Burst) low amplitude off off on on off US(Burst) high amplitude off on on off


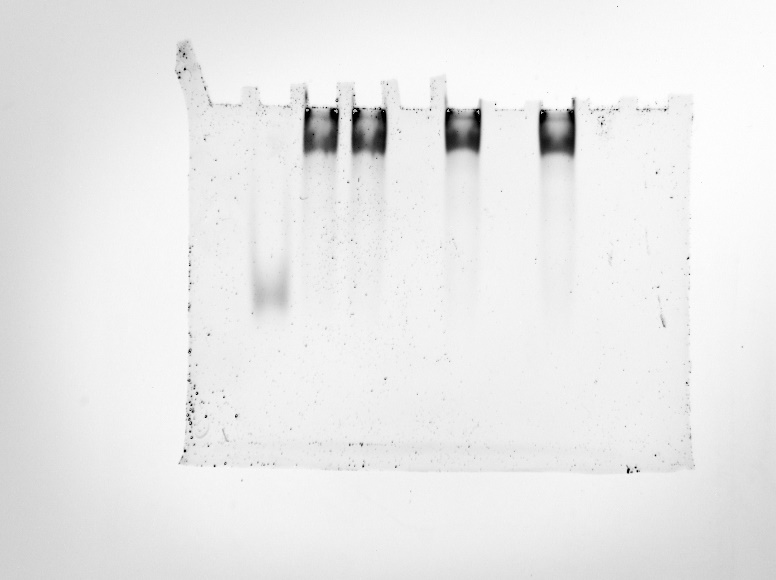




1 2 3 4 5 6 7 8 9

1. **D)**

ASO rHA-cODN(PO)/ASO ASO rHA-cODN(PO)/ASO

Time 0 1 6 16 Time 0 1 6 16

US(continuous) low amplitude off off on on off US(continuous) high amplitude off off on on off


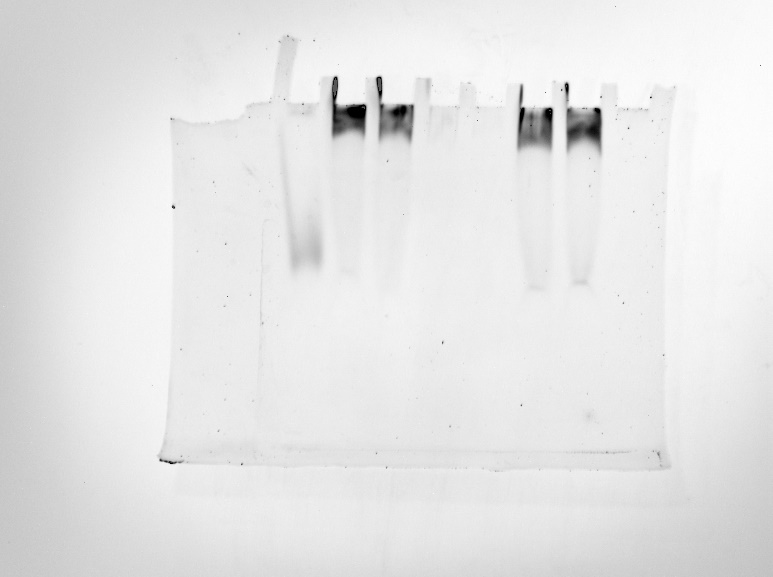

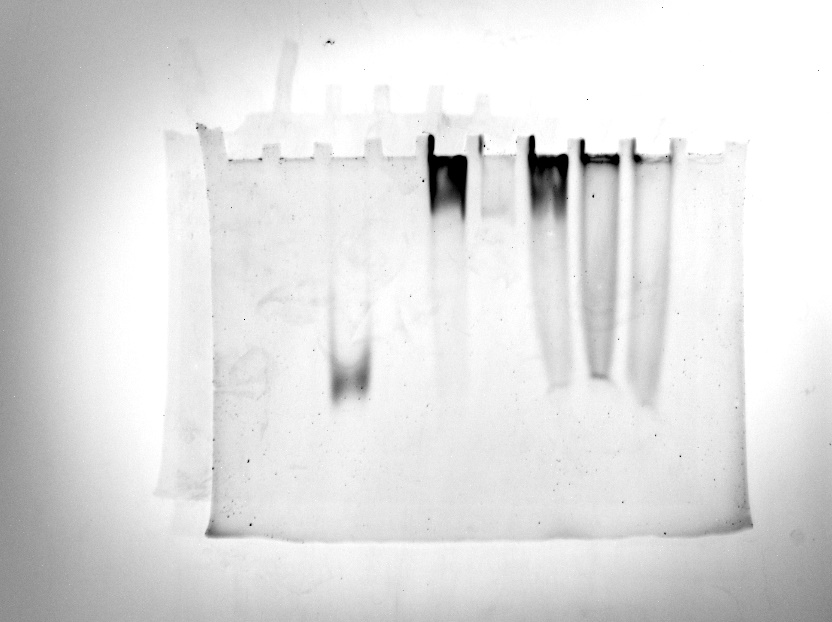


10 11 12 13 14 15 16 17 18 19

Figure S 4 Full gel image of Figure 1. SYBR Gold staining of 8% native PAGEs for the rHA-cODN(PO)/ASO biomolecular assembly after US exposure at different modes. A & B) Low and high amplitudes burst mode respectively. C & D) Low and high amplitudes continuous mode respectively. Lanes 1, 10 & 15: ASO, lanes 2-5, 6-9, 11-14 & 16-19: rHA-cODN(PO)/ASO biomolecular assembly.

1. **B)**

SYBR Gold SYBR Green

ASO rHA-cODN Not applicable ASO rHA-cODN Not applicable

(PO)/ASO in manuscript (PO)/ASO in manuscript

US off off on US off off on


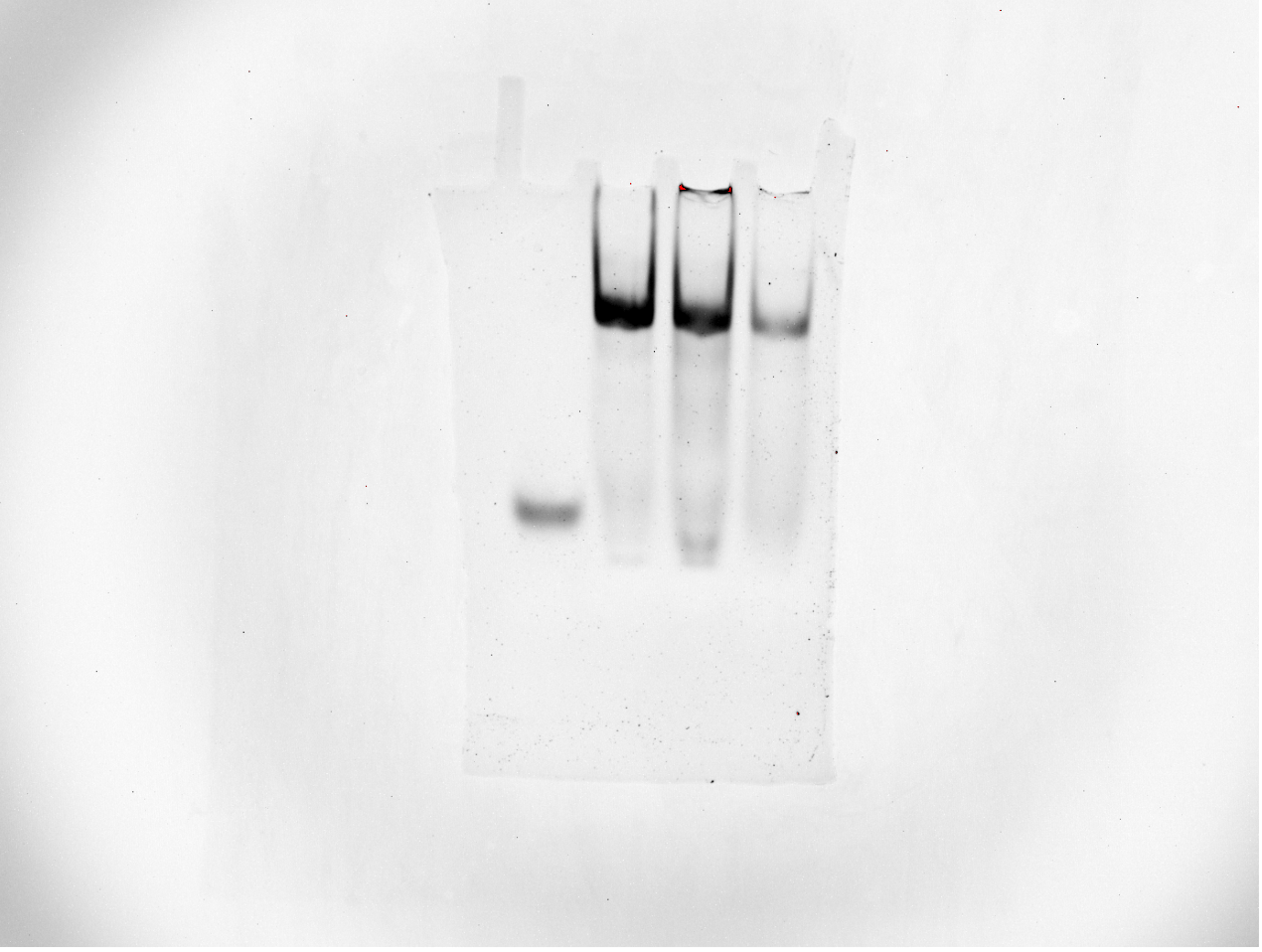

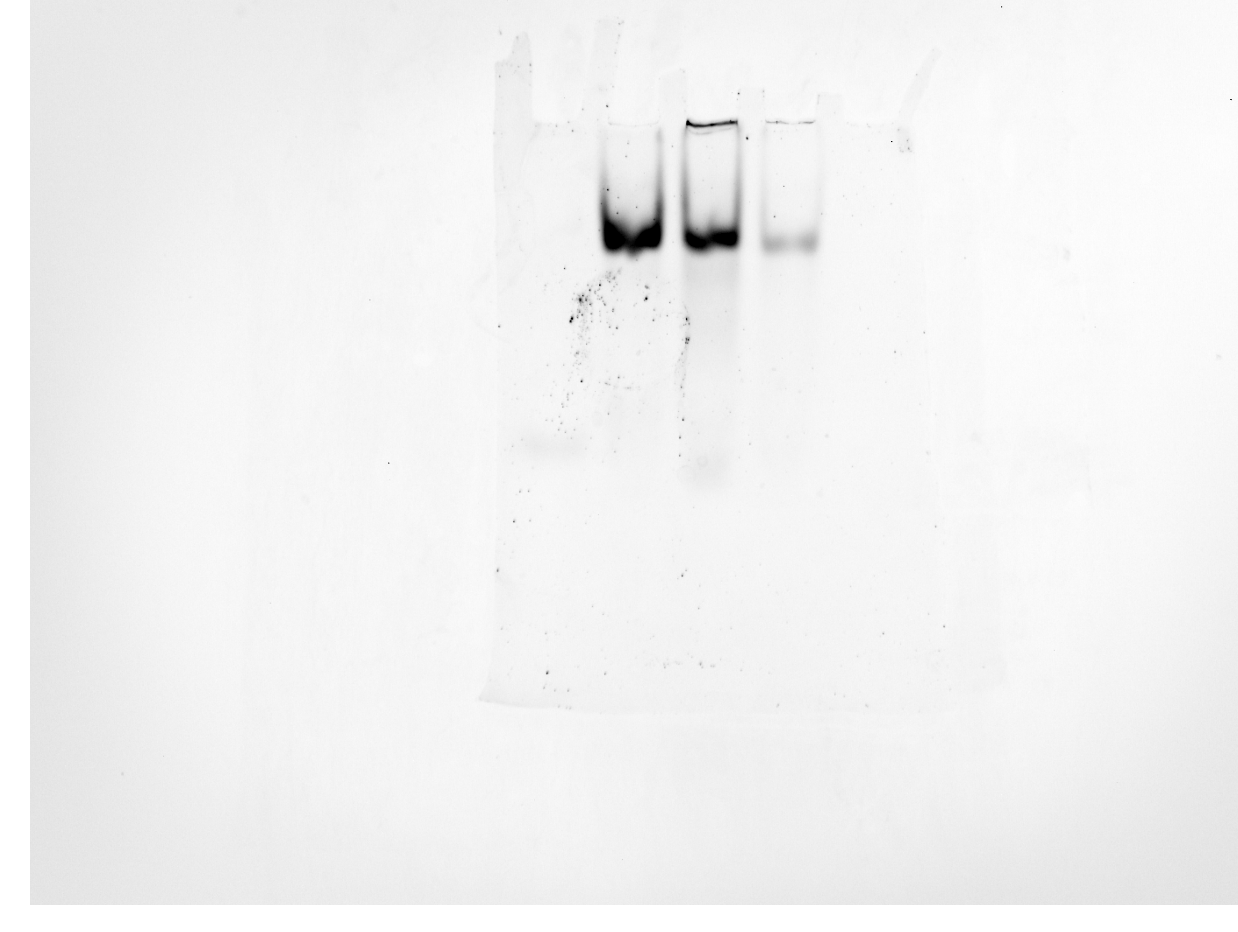


1 2 3 4 5 6

Figure S 5 Full gel image of Figure 2. US-mediated ASO release (low amplitude continuous mode for 30 min). A & B) SYBR Gold and SYBR Green imaging of 8% native PAGE respectively, lanes 1 & 2: ASO, lanes 2,3,5 & 6: rHA-cODN(PO)/ASO.

**A) B)**

HPLC purified ASO rHA-cODN ASO rHA-cODN

rHA-cODN rHA-cODN ASO (PO)/ASO (PO)/ASO

(PO)/ASO (PS)/ASO

Not applicable in manuscript DNase - - + - - + Not applicable in manuscript


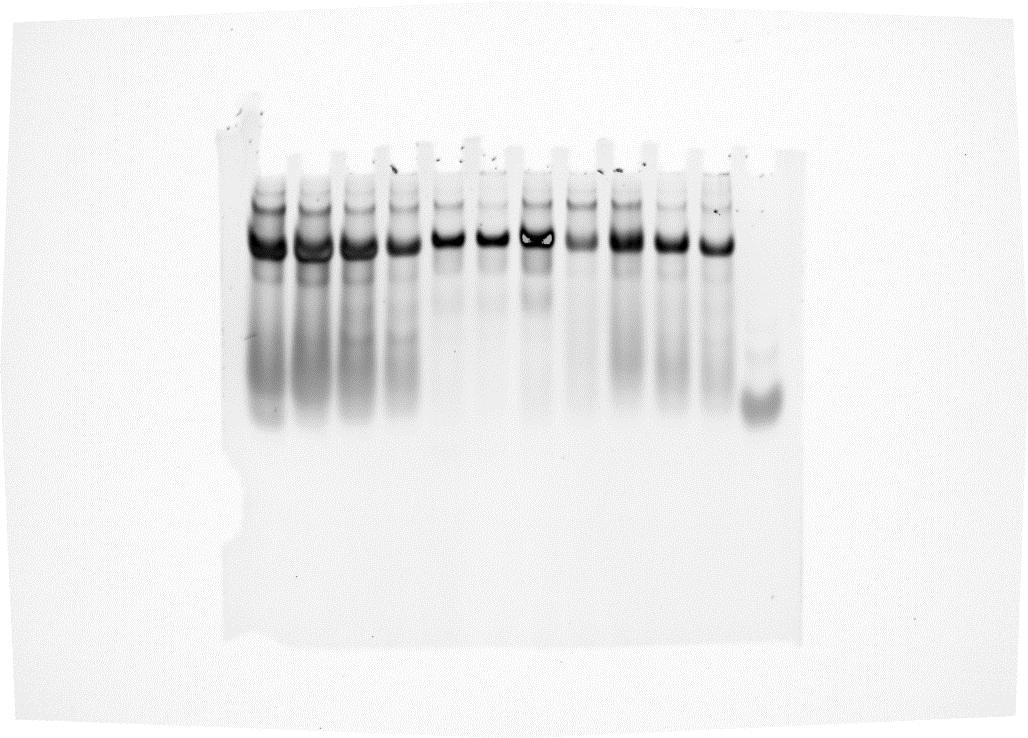

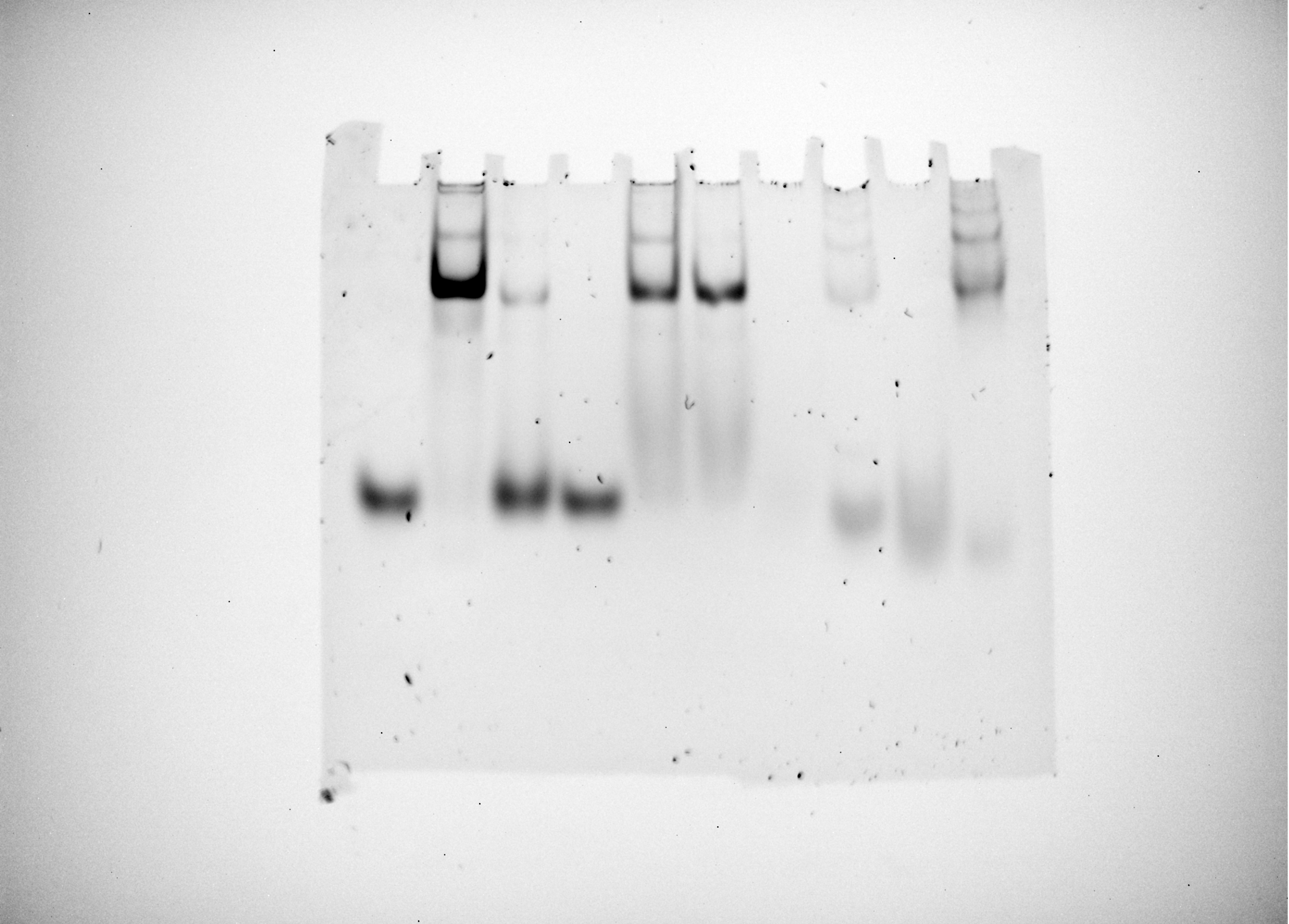


1 2 3 4 5 6 7 8 9

**C) D)**

ASO rHA-cODN(PO)/ASO ASO rHA-cODN(PS)/ASO

US (continuous low amplitude) off off on off off on


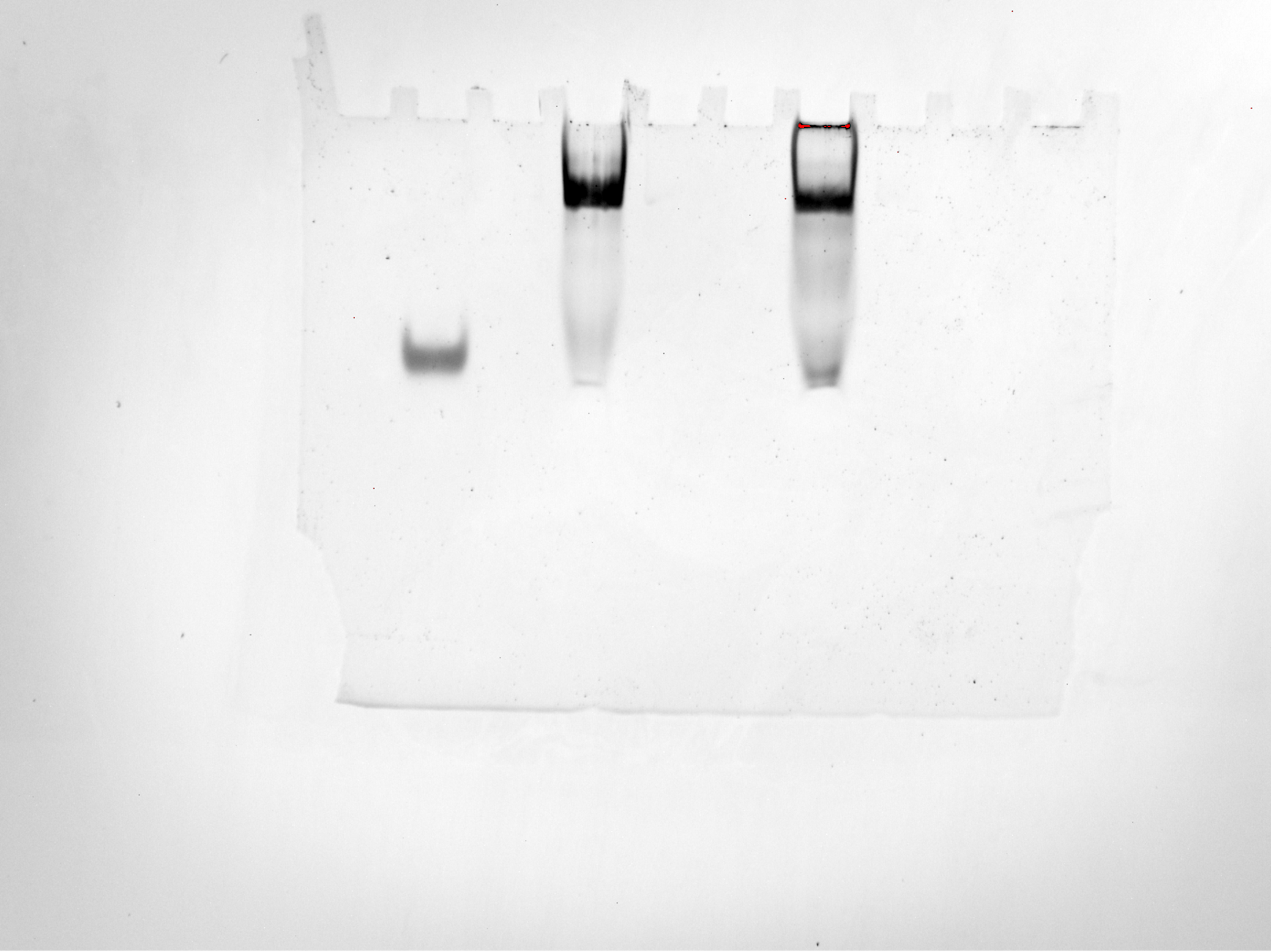

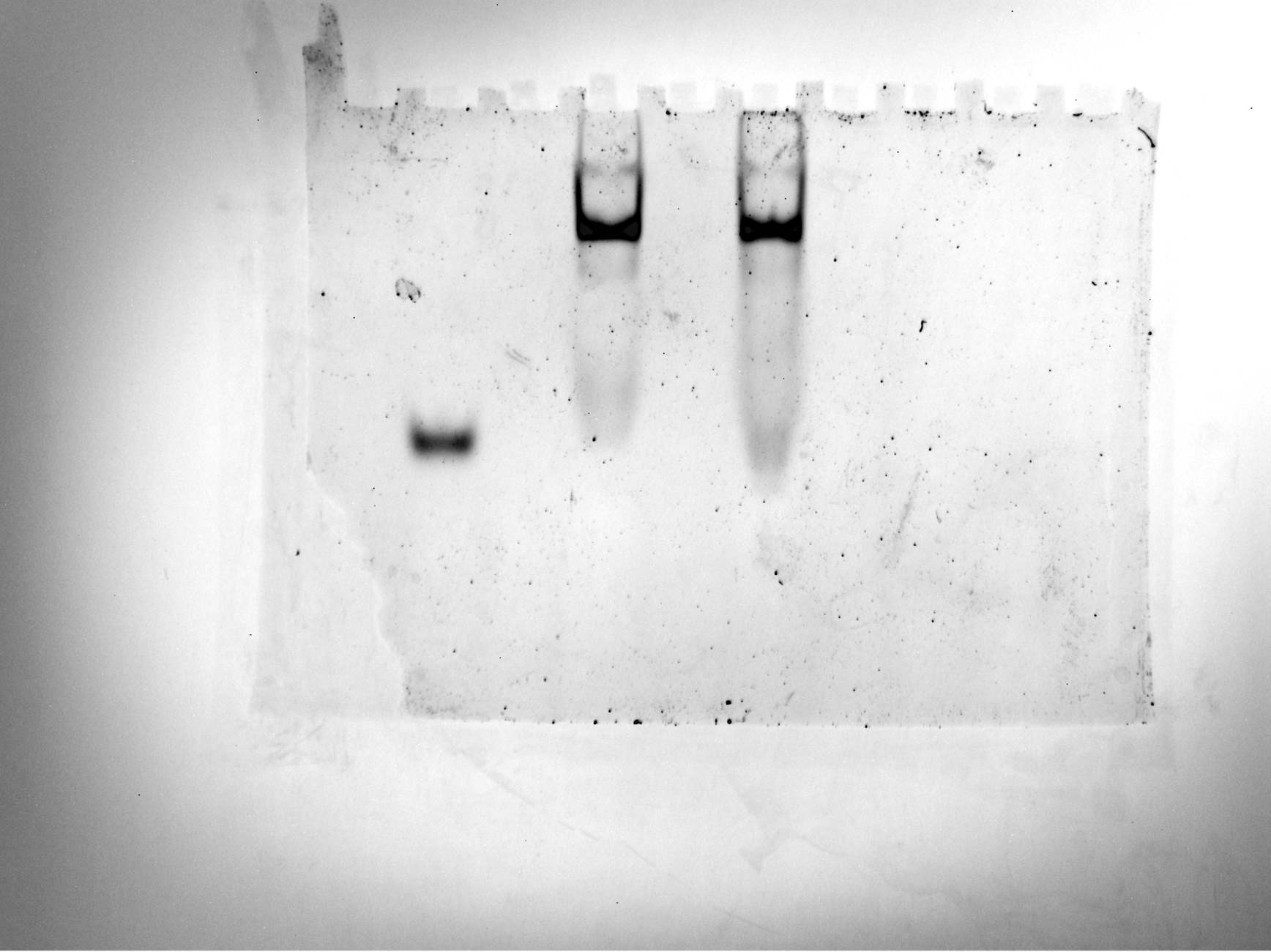


10 11 12 13 14 15

Figure S 6 Full gel image of Figure 3. PAGE showing the different biomolecular assemblies after HPLC purification and effect of DNase and US (Low amplitude continuous mode for 30 min) on the ASO release. A) SYBR Gold staining of 8% native gel of the different assemblies. Lane 1: rHA-cODN(PO)/ASO, lane 2: rHA-cODN(PS)/ASO, lane 3: ASO. B) SYBR Gold staining of 8% native gel showing the effect of DNase on the release of ASO from different assemblies. Lane 4&7: ASO, lane 5 & 6: rHA-cODN(PO)/ASO, lane 8 & 9: rHA-cODN(PS)/ASO. C & D) SYBR Gold staining of 8% native gel showing the effect of US on rHA-cODN(PO)/ASO and rHA-cODN(PS)/ASO respectively. Lane 10 & 13: ASO, lane 11 & 12: rHA-cODN(PO)/ASO, lane 14 & 15 : rHA-cODN(PS)/ASO.
